# Technique of interdigitating flaps for repair of abnormal origin of right pulmonary artery from ascending aorta

**DOI:** 10.1016/j.xjtc.2024.03.021

**Published:** 2024-04-05

**Authors:** Gananjay G. Salve, Danish A.K. Memon, Veeresh Manvi, Nidhi G. Manvi, Mohan D. Gan, Richard Saldanha

**Affiliations:** a Department of Cardiovascular & Thoracic Surgery, KLE's Dr Prabhakar Kore Hospital & Medical Research Centre, Belgaum, Karnataka, India; b Department of Paediatric Cardiology, KLE's Dr Prabhakar Kore Hospital & Medical Research Centre, Belgaum, Karnataka, India

**Figure:**
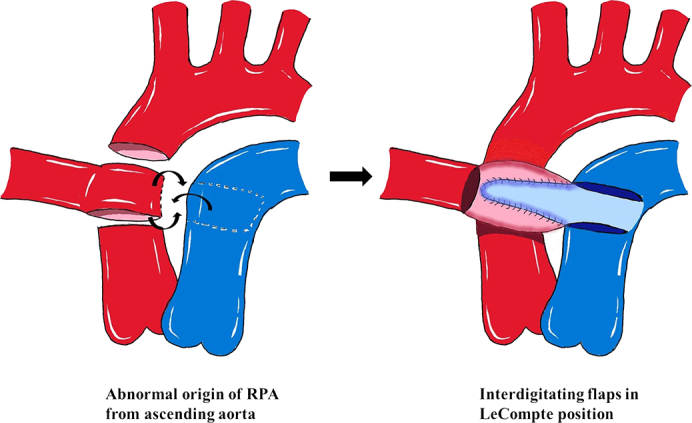
Diagram of interdigitating pulmonary artery flap and aortic flaps in the LeCompte position.

**Central Message**

The novel technique of interdigitating autologous great arterial flaps is an effective option in the surgical armamentarium for repair of AORPA from the ascending aorta.

Abnormal origin of the right pulmonary artery (AORPA) from the ascending aorta (right hemitruncus) is a rare congenital cardiovascular anomaly presenting in infancy with 70% first-year mortality if not operated.^1^ Early primary surgical repair is the treatment of choice with good outcomes.^2^^,^^3^

We describe a novel technique of interdigitating flaps of autologous great arterial walls to implant AORPA onto the main pulmonary artery.

## Case Report

**Figure 1:**
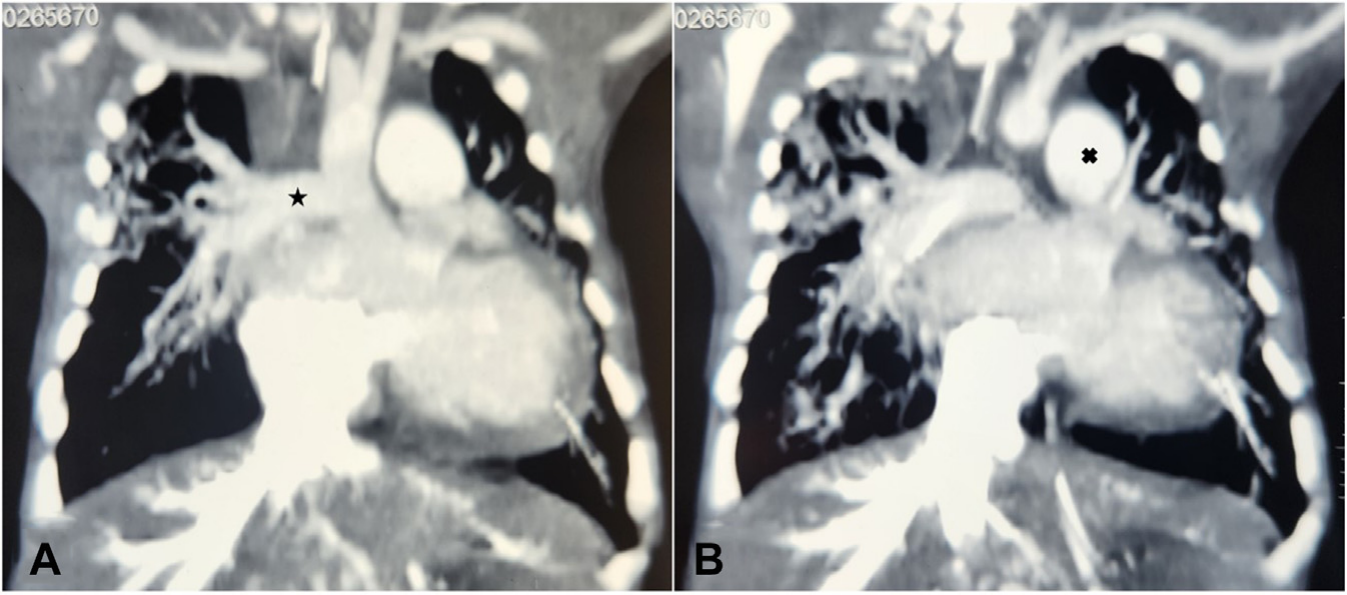
A, Contrast-enhanced computed tomography image in coronal section showing abnormal origin of the RPA from the ascending aorta. B, Contrast-enhanced computed tomography image in coronal section showing main pulmonary artery continuing as left pulmonary artery.

A 27-day-old male child, weighing 2.6 kg, with severe respiratory distress, was diagnosed with AORPA from ascending aorta, confirmed on contrast-enhanced computed tomography scan (Figure 1).

**Figure 2:**
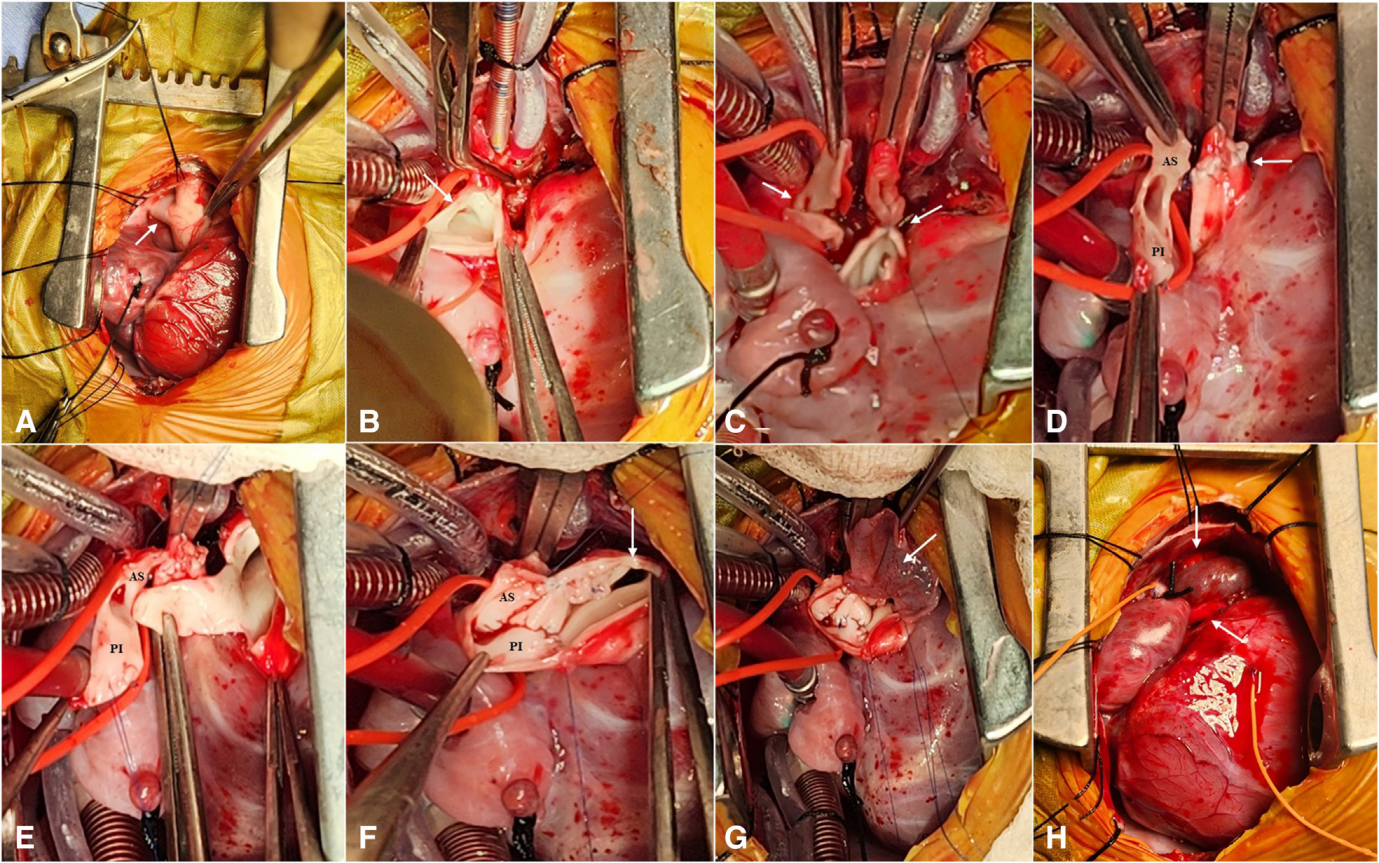
A. Intraoperative photograph after pericardiotomy showing the RPA arising from ascending aorta (*arrow*). B, Transverse aortotomy has been done showing the origin of RPA (*arrow*). C, The RPA has been harvested along with the flaps of the native aortic wall (*left arrow*). Direct anastomosis of ascending aorta has been commenced (*right arrow*). D, Origin of RPA with its anterosuperior flap and posteroinferior flap. *Arrow* is pointing toward the completed aortic reconstruction. E, Transverse main pulmonary artery flap has been created (held in the forceps) that opens like a trapdoor toward the RPA origin to interdigitate between the 2 aortic wall flaps (anterosuperior, posteroinferior). F, Completed anastomosis of the interdigitating flaps using 6-0 polypropylene continuous sutures. The *arrow* is pointing toward the origin of left pulmonary artery. G, Autologous treated pericardial patch is being used to reconstruct the anterior wall of the RPA (*arrow*). H, Completed reconstruction of the RPA showing anterosuperior flap (*vertical arrow*) and posteroinferior flap (*oblique arrow*).

Surgical repair was performed through a median sternotomy. The right pulmonary artery (RPA) (Figure 2, *A*) and aortopulmonary groove were dissected, and both branch pulmonary arteries were looped. Cardiopulmonary bypass was established with aorto-bicaval cannulation. Cardioplegic cardiac arrest was achieved, and transverse aortotomy was performed just above the origin of the RPA (Figure 2, *B*). A transverse ring of ascending aortic wall was harvested adjoining the origin of the RPA, and the ring was divided exactly opposite to the origin to create 2 equal flaps of aortic wall. The aorta was reconstructed by direct anastomosis (Figure 2, *C* and *D*).

Aortic flaps attached to the RPA origin were now slightly tilted to form an anterosuperior (anterior) flap and a posteroinferior (posterior) flap. A transverse flap was incised on the anterior wall of main pulmonary artery like a trapdoor opening medially toward the RPA origin (Figure 2, *E*). This pulmonary artery flap was sutured in between the 2 aortic flaps, using 6-0 polypropylene continuous sutures, so that the great arterial wall flaps interdigitate among each other (Figure 2, *F*). This formed the posterior two-thirds of the RPA wall, anterior to the reconstructed ascending aorta. The anterior wall of RPA was reconstructed with treated autologous pericardium (Figure 2, *G* and *H*).

**Figure E1:**
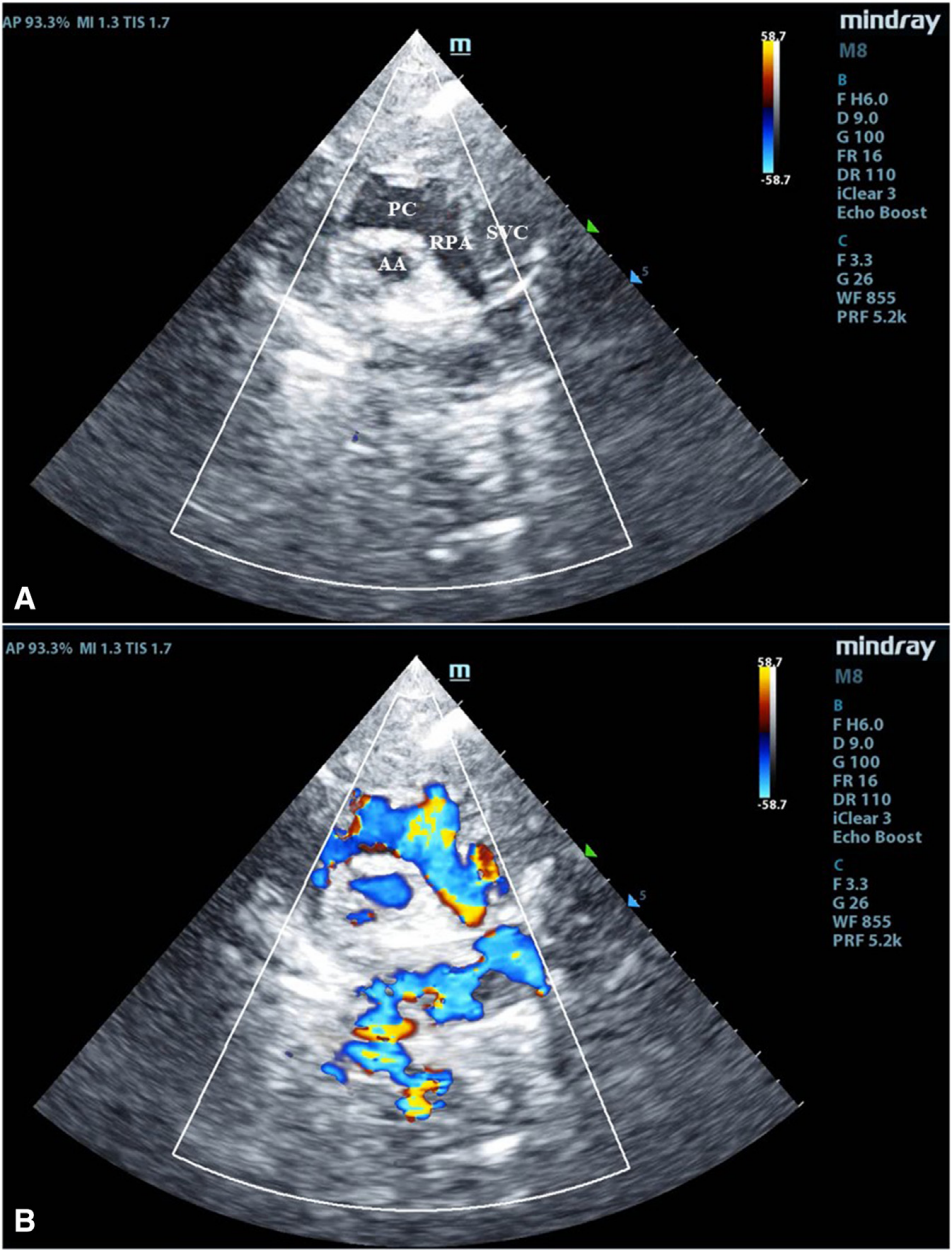
A, Two-dimensional echocardiography image in short-axis view showing the pulmonary confluence along with the origin of the RPA, anterior to the ascending aorta. Superior vena cava seen anterior to the distal RPA. B, Color Doppler image of the same view showing laminar flow in the RPA.

**Figure E2:**
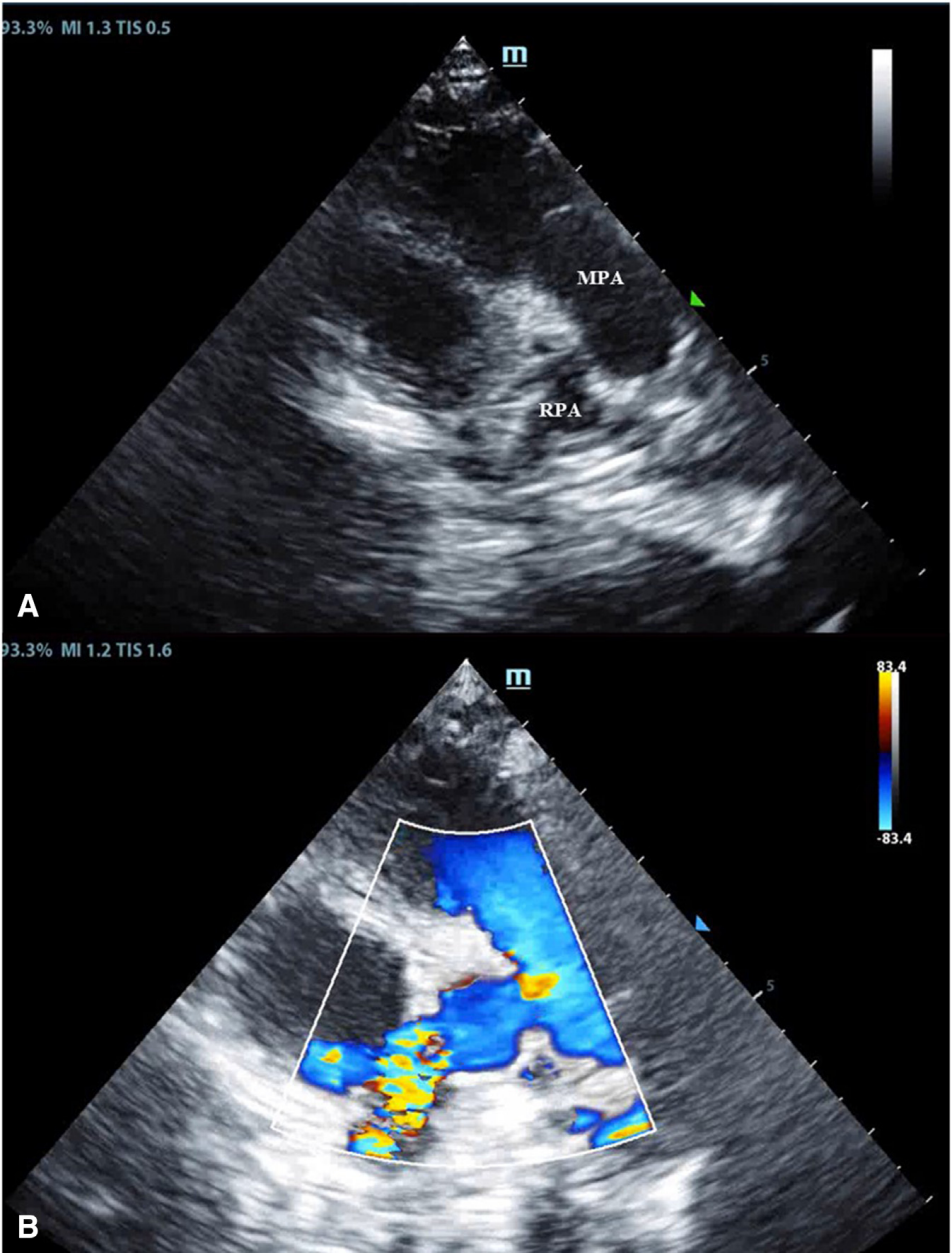
A, Two-dimensional echocardiography image at 4-month follow-up in short-axis view showing the main pulmonary artery and RPA. B, Color Doppler image of the same view showing laminar flow in the RPA.

Cardiopulmonary bypass was weaned uneventfully, and the patient was shifted out with a closed chest. Heparin infusion was started 8 hours after surgery, and antiplatelet therapy was administered the next day. He was discharged on low-dose antiplatelet therapy. Postoperative and follow-up echocardiography showed good flow in the RPA (Figures E1 and E2).

## Discussion

Several surgical techniques have been described for repair of AORPA from the ascending aorta.^2^ Posterior direct anastomosis and pericardial patch anteriorly (technique III)^2^ always have a possibility of compression by the ascending aorta from the front. Techniques IVA and IVB^2^ create aortic flaps and directly anastomose it to the pulmonary artery with or without using a pulmonary artery flap. These techniques are well described, but it is not always possible to achieve a direct anastomosis without stretch or luminal compromise.

In our technique, the posterior two-thirds or more of the RPA anastomotic lumen is native tissue. This preserves its growth potential. As the flaps move toward each other, there is no stretch on the reconstructed anastomosis. Also, direct anastomosis of the aorta behind the reconstructed RPA ensures that the ascending aorta is pushed posteriorly, avoiding compression on RPA.

There is always an option of using a prosthetic interposition graft,^4^ but at the cost of a definitive redo surgery. Early direct anastomosis without use of any graft material has been recently reported with good outcomes.^5^

We manipulate the anterior flap to the anterosuperior position and the posterior flap to the posteroinferior position, possibly causing a clockwise rotation of the RPA lumen to approximately 45 degrees. We believe that as the rotation is distributed all along the length of RPA, it does not introduce significant obstruction. This is evident by laminar blood flow in the RPA noted in immediate postoperative and follow-up echocardiography studies (Figures E1 and E2). We recommend regular long-term follow-up imaging to ensure unobstructed flow in the RPA.

## Conclusions

We conclude that our novel technique of repairing AORPA from the ascending aorta is simple, reproducible, and tension-free, preserving the future growth potential of the vessel.

## Conflict of Interest Statement

The authors reported no conflicts of interest.

The *Journal* policy requires editors and reviewers to disclose conflicts of interest and to decline handling or reviewing manuscripts for which they may have a conflict of interest. The editors and reviewers of this article have no conflicts of interest.
